# Characterizing the Aging Process of the Human Eye: Tear Evaporation, Fluid Dynamics, Blood Flow, and Metabolism-Based Comparative Study

**DOI:** 10.1155/2022/2805402

**Published:** 2022-03-24

**Authors:** Md Ashiqur Rahman, Mamun Rabbani, Md Hasan Maruf, Aminul Islam, A. S. M. Shihavuddin

**Affiliations:** ^1^Department of EEE, Green University of Bangladesh (GUB), 220/D Begum Rokeya Sarani, Dhaka 1207, Bangladesh; ^2^Department of Biomedical Physics and Technology, University of Dhaka, Dhaka -1000, Bangladesh; ^3^Technical University of Denmark, Denmark; ^4^Department of CSE, Independent University Bangladesh (IUB), Plot 16, Aftab Uddin Ahmed Rd, Dhaka 1229, Bangladesh

## Abstract

Eye temperature and intraocular pressure are two measurable parameters that can be monitored as a health index with aging. Deviations from the normal range of intraocular pressure and temperature lead to the formation of many diseases. This study has been carried out to evaluate the relations between the physiological and anatomical changes of the eye with aging using mathematical modeling. 2D computer-aided design of the human eye has been developed for two major groups: 21 to 30 years and 41 to 50 years. The computer simulation has been carried out to determine the effects of physiological changes of tear evaporation, fluid dynamics, blood flow, and metabolism of eye tissues with aging. The simulation has been carried out in the standing and the supine position of a human body. The rate of temperature change is – 0.0075 K per year in the standing position and – 0.007 K per year in the supine position because of the modeled anatomical and physiological effects. All the three simulation parameters of this study, the temperature of the human eye, the intraocular pressure, and the aqueous humor flow velocity, have been compared with the recent practical and simulation-based experiments to validate our results.

## 1. Introduction

Physiology is the study of the functionalities of the parts of the human body. Mathematical modeling of the physiology of the human body can be helpful to estimate the aging and the progress of diseases [1]. The human eye is the sensory organ that creates the sense of vision. Due to the lack of natural protection, through the eyes, external particles can enter the human body to exert adverse effects on natural activities of the human body and thus cause fatal diseases [2]. Globally, millions of people are suffering from different ocular diseases like glaucoma, ocular hypertension, and cataract. The root causes of many of the diseases are still unknown. Diseases are more likely to occur with aging [3, 4]. The anatomical and physiological changes of the human eye over the years from the birth of a person cause the ocular temperature and the intraocular pressure to vary with age. A mathematical model of ocular aging can help us to estimate the changes with aging compared to the diseases. In this work, we have developed a method to synthetically model the temperature profile and the fluid dynamics of aqueous humor flow of the human eye with aging and generated an estimated base model. Based on the estimated base model, we can effectively detect anomalies from natural data and also at the same time simulate adverse conditions exploring the root cause of anomalies.

Computer simulation can aid to visualize and predict the temperature profile of the human eye. The sensitivity of the human eye to any external contact makes it difficult to measure the temperature of the eye tissues [5]. Using contactless techniques, it is possible to obtain the temperature of the ocular surface accurately, but it cannot provide much information on the temperature of internal tissues of the eye. The experiments on the living animal eyes do not portray the physiology of the human eye properly [6]. The advancement of computing technology has made it possible to apply numerical methods to the complex geometry of the human eye to develop the temperature profile. Computer simulation of ocular thermodynamics has been studied extensively over the years. Research methodologies of the previous works include 1D, 2D, and 3D geometry of eye and different numerical techniques like finite element method (FEM) and boundary element method [7–11].

Literature review shows that age-related temperature profiling is a study area to be explored in detail. Practical experiments have revealed that ocular surface temperature decreases with age. The rate of decline of ocular surface temperature is – 0.038 K per year according to the study based on infrared imaging by Acharya et al. [12]. To explore the reasons for temperature decrease with age, simulation-based temperature profiling has been carried out by Samaras [13] and Bhandari et al. [14]. The study of 2014 has modeled the age-related ocular geometry and the functional changes with tear evaporation. The study of 2020 has extended the work by including the age-related aqueous humor flow. But the effects of ocular metabolism and blood perfusion on ocular aging have not been modeled in both the previous studies. Practical experiments on ocular temperature have hypothesized that reduction in metabolism and blood flow is one of the factors of temperature decline with age. The effects of blood perfusion on ocular temperature, studied by Rafiq and Khanday (2016), show that the ocular temperature decreases for reduced blood flow. But it has not incorporated the aging effects or the fluid dynamics simulation [15]. Thus, modeling the age-dependent changes of blood perfusion and metabolism in the ocular tissues is an area of interest to be explored yet.

The human eye has the fluid-filled regions of aqueous humor (AH) and vitreous humor (VH). The dynamics of the fluid-filled regions especially the aqueous humor is an important indicator of a healthy eye. Deviation from the normal aqueous humor flow results in ocular diseases like glaucoma [16]. Simulation-based ocular fluid dynamics has been studied in recent years and a comparative summary of these works is given in Table 1. Navier-Stokes equation of fluid dynamics has been applied to study the AH flow in these studies. The research works are distinct from each other for applying different boundary conditions in numerical simulations. Darcy's law is one of the best approximate mathematical models of the fluid flow through the porous structure of trabecular meshwork [17, 18]. Darcy's law relates the pressure drop across a porous material with the velocity of the fluid flow. The literature review of the recent research works, given in Table 1, shows that the age-related aqueous humor flow simulation applying Darcy's law is yet to be explored.

In this study, simulation applying numerical methods has been carried out to model the aging effects on ocular anatomy and physiology. The simulation incorporates the age-related physiological changes due to metabolism, blood flow, tear evaporation, and aqueous humor flow. The anatomical changes are modeled in the developed 2D computer-aided design of the human eye. This study has developed a more detailed computer-aided design of the human eye including ten ocular tissues. The previous studies on the simulation of ocular aging in 2014 and 2020 have six and nine homogenous regions in their designs, respectively, where the retina and choroid have been neglected [13, 14]. The retina and choroid have been modeled as two separate homogenous regions in the CAD model of this study as the thermal properties of these tissues are different [21]. The flow through the trabecular meshwork has been modeled using Darcy's law to simulate the age-dependent changes of intraocular pressure and aqueous humor fluid dynamics.

## 2. Methods and Materials

### 2.1. Anatomy of the Human Eye with Aging

**Figure 1:**
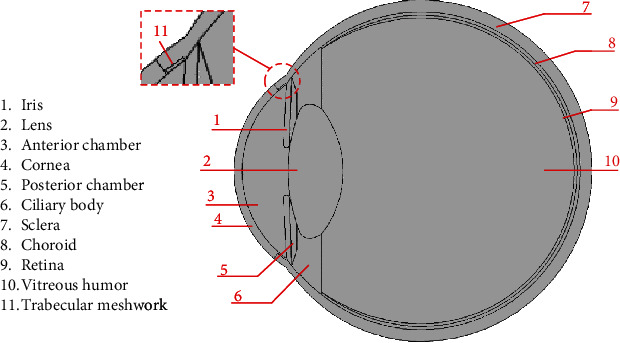
Homogenous regions of the developed 2D CAD model of the human eye.

**Figure 2:**
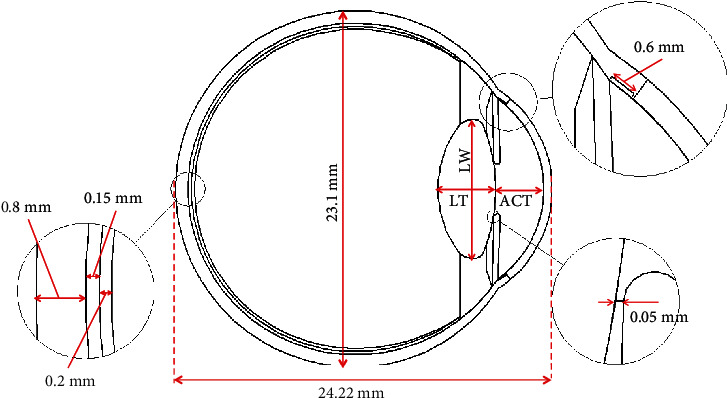
2D geometry of the computer-aided design of human eye.

In this study, 2D computer-aided designs (CAD) of two age groups (young: 21 to 30; elder: 41 to 50 years) have been developed using SolidWorks. The design has eleven homogenous regions: cornea, anterior chamber (AC), posterior chamber (PC), vitreous humor (VH), lens, iris, ciliary body (CB), trabecular meshwork (TM), sclera, choroid, and retina. The homogenous regions of the developed human eye model are shown in Figure 1. The human eye CAD model has been developed based on dimensions shown in Figure 2. In this design, the length of the eyeball is 24.22 mm horizontally and 23.1 mm vertically. The thickness of the cornea is 0.54 mm. The computer-aided design (CAD) of the human eye has been developed following the dimensions of literature data [21, 24, 25]. Human eye anatomy changes with age. The lens of the human eye grows due to aging. The width and the thickness of the lens increase with age. Consequently, the depth of the anterior chamber and the distance between cornea and lens decrease with age [26, 27]. In this study, the age-related changes, shown in Table 2, have been modeled in the 2D CAD design of the human eye.

### 2.2. Physiology of the Human Eye with Aging

The tear is produced at the lacrimal apparatus, and blinking of the eyelid spreads it over the external surface of the eye. The drainage of tears is through the nasolacrimal duct [28, 29]. Evaporation of lacrimal fluid or tear contributes to the thermodynamics of the eye significantly. As tear evaporation causes heat transfer between the cornea and external environment [11], Guillon and Maissa have studied the rate of tear evaporation of two healthy groups using evaporimeter: young (28.1 ± 7.3years old) and elder (53.7 ± 7.8years old) [30]. The collected experimental data of tear evaporation, shown in Table 3, have been applied to model the young and the elder group in the simulation of this study. Aqueous humor (AH) is secreted from the ciliary body into the posterior chamber. Then, the AH fluid enters the anterior chamber flowing through the gap between the iris and lens. Finally, it drains through the trabecular meshwork (TM) [31]. The rate of AH production decreases with age. The rate of decline is about 2.4% per ten years. In this study, the aqueous humor flow rates of the two age groups are shown in Table 3.

Blood perfusion is the volumetric rate of blood flow through a unit volume of tissue of the human body. Studies on ocular blood perfusion show that the blood perfusion through the choroid and retina reduces with age. Ocular blood flow of healthy persons, aged 21 to 30 years, has been measured using magnetic resonance imaging (MRI) by Khanal et al., where the blood perfusion through choroid and retina has average 0.01298 ml (ml^−1^s^−1^) and standard deviation 4.97 × 10^−3^ml(ml^−1^s^−1^) [32]. Blood flow through the human eye decreases about 10% per decade which has been observed in another MRI-based study by Emeterio Nateras et al. [35]. So, in our simulation, the blood perfusion rate in the choroid and retina, shown in Table 3, has been modeled based on these two experimental studies. The blood perfusion rate of the iris in Table 3 has also been collected from the literature survey [33, 34].

Metabolism is a regular activity of any living tissue. The living cells of the human body produce energy through biochemical reactions using the oxygen supplied by the blood flow. Basal metabolism of a tissue is the volumetric energy expenditure expressed in Wm^−3^. Hirata et al. [34] have estimated the basal metabolism rate of the ocular tissues to fit the temperature distribution of healthy eyes mathematically. The age-dependent data of ocular metabolism is not available in the literature. Metabolism of any tissue depends on the blood flow and they are almost proportionally related [33]. As the blood perfusion rate of ocular tissues declines about 10% per decade, the metabolism with aging has been reduced by the same percentage in this study as shown in Table 3.

### 2.3. Governing Equations of Heat Transfer

The equation of heat transfer in the human eye is shown in equation (1). The equation is known as the Pennes bioheat transfer equation, and it has been applied to study the temperature profile of the human eye by researchers over the past decades [36–38]. In this study, the steady-state temperature profile has been simulated using this equation.

(1) $$\begin{array}{c} \rho_{a}c_{a}\left(\overset{⟶}{v}.\nabla T\right)+\nabla \left(-\alpha \nabla T\right)=Q_{\text{blood}}+Q_{m}. \end{array}$$

Convective and conductive heat transfer in the human eye is governed by the first and second terms of the left-hand side of this equation, respectively. In this equation, *T* is the temperature of ocular tissues, and ∇*T* represents the change of temperature spatially in the simulation. Convective heat transfer occurs due to the flow of aqueous humor fluid in the eye. The velocity vector of aqueous humor fluid flow is $\overset{⟶}{v}$. *ρ*_*a*_ and *c*_*a*_ represent the density and the heat capacity at the constant pressure of aqueous humor, respectively. The conductive heat transfer occurs in all tissues of the human eye. *α*  represents the thermal conductivity of ocular tissue. *Q*_blood_ and *Q*_*m*_ represent heat generation due to blood flow and metabolic activities in the eye, respectively. *Q*_blood_ can be further expressed as [21]

(2) $$\begin{array}{c} Q_{\text{blood}}=\omega_{b}c_{b}\rho_{b}\left(T_{b}-T\right), \end{array}$$

where *ω*_*b*_ is the blood perfusion rate, *c*_*b*_ is the heat capacity of blood at constant pressure, *ρ*_*b*_ is the density of blood, and *T*_*b*_ is the blood temperature. Simulation parameters of blood perfusion, *ω*_*b*_, and the metabolic heat generation, *Q*_*m*_, have been described in Section 2.2. The blood temperature in this study is 310 K. Blood has a density of 1050 kgm^−3^, and the heat capacity at constant pressure is 3600 JKg^−1^ K^−1^ [14].

The boundary condition at the sclera is due to the blood circulation at the choroid [39]. The generated heat spreads due to convection. The governing equation of this boundary condition is shown in equation (3), where *n* is the normal direction to the surface boundary. *h*_*b*_ is the convection coefficient of blood which is equal to 65 Wm^−2^K^−1^ [14].

(3) $$\begin{array}{c} \alpha \frac{\partial T}{\partial n}=h_{b}\left(T-T_{b}\right). \end{array}$$

The cornea is an open boundary where the cooling or the loss of heat occurs [5]. The heat transfer at the cornea involves three processes including radiative heat loss, convective heat transfer, and heat loss due to tear evaporation. The boundary condition is governed by

(4) $$\begin{array}{c} \alpha \frac{\partial T}{\partial n}=h_{\text{amb}}\left(T-T_{\text{amb}}\right)+\sigma \epsilon \left(T^{4}-T_{\text{amb}}^{4}\right)+E, \end{array}$$

where the term on the left-hand side represents the heat flow in the normal direction to the corneal surface. The convection of heat between the cornea and the surrounding environment is modeled by the first term of the right-hand side. The ambient convection coefficient, *h*_amb_, is 10 Wm^−2^K^−1^ [14]. The ambient temperature, *T*_amb_, is 296 K. The second term represents the radiative heat transfer between the corneal surface and the external environment. The Stefan–Boltzmann constant, *σ*, is equal to 5.67 × 10^−8^Wm^−2^K^−4^. The emissivity of the cornea, *ϵ*, is 0.975 in the simulation [14]. The third term, *E*, represents the phenomenon of tear evaporation which is shown in Table 3.

### 2.4. Governing Equations of Aqueous Humor Flow

The flow of aqueous humor (AH) inside the anterior chamber is modeled using the Navier-Stokes equation for incompressible fluid under steady-state conditions [19]. The transposed velocity gradient has been neglected as it has negligible effects if the viscosity is constant.

(5) $$\begin{array}{c} \rho_{a}\left(\overset{⟶}{v}.\nabla \right)\overset{⟶}{v\;}=-\nabla p+\nu \nabla^{2}\overset{⟶}{v}+\overset{⟶}{F}+\rho_{a}\overset{⟶}{g}, \end{array}$$

(6) $$\begin{array}{c} \nabla .\overset{⟶}{v}=0. \end{array}$$

where *p* is the pressure inside the anterior chamber, *ρ*_*a*_ is the density of AH, *ν* is the dynamic viscosity of AH, $\overset{⟶}{v}$ is the velocity vector of AH flow, and $\overset{⟶}{g}$ is the gravitational acceleration. There is a temperature gradient from the posterior surface of the cornea to the anterior surface of the lens or the opening of the pupil. Convection of aqueous humor results from this temperature gradient [22]. To model this effect, the Boussinesq approximation, $\overset{⟶}{F,}$ has been included in the simulation as shown in

(7) $$\begin{array}{c} \overset{⟶}{F}=\rho_{a}\overset{⟶}{g}\lambda \left(T-T_{\text{ref}}\right), \end{array}$$

where *λ* = 0.000337K^−1^ is the thermal expansion coefficient and *T*_ref_ = 310 K is the reference temperature in the AH flow simulation.

**Figure 3:**
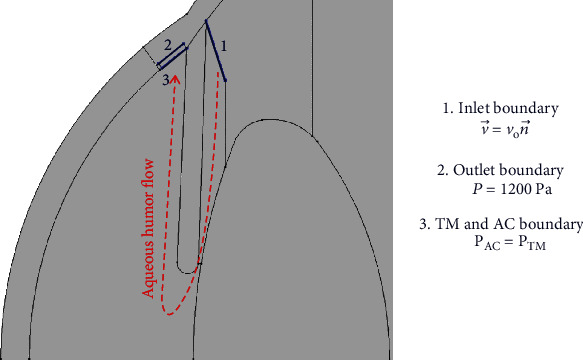
Boundary conditions and flow direction of aqueous humor.

Trabecular meshwork (TM) is a porous medium. 80 to 90% outflow of aqueous humor is through the trabecular meshwork [18]. Outflow through TM is simulated in this study as the pressure gradient across TM maintains the intraocular pressure. The rest of the outflow through uveoscleral path has not been simulated due to its negligible effects on intraocular pressure [40]. Darcy's law and Carmen-Kozeny equation have been successful to estimate the porous characteristics of the trabecular meshwork. Darcy's law relates the change of pressure across the porous media with the flow velocity. The Carmen-Kozeny equation relates the permeability with the porosity of porous media. Porosity defines the percentage of the porous region in porous media. Permeability is related to the conductivity of fluid flow in a porous structure [17, 41]. The more permeability, the less resistance to the fluid flow. In the simulation, TM is modeled as a porous homogenous medium, and the governing equation of flow through TM is commonly known as Darcy's law.

(8) $$\begin{array}{c} \overset{⟶}{v}=-\frac{k}{\nu }\nabla p, \end{array}$$

where *k* is the permeability of trabecular meshwork and *ν* is the dynamic viscosity of aqueous humor fluid. The permeability and the porosity, ∅ of TM, are related by the Carman-Kozeny equation as shown in [42]

(9) $$\begin{array}{c} k=\frac{D\emptyset^{3}}{150{\left(1-\emptyset \right)}^{2}}, \end{array}$$

where *D* is the diameter of the pore in the trabecular meshwork. In this study, considering *D* = 8 × 10^−6^ m, the permeability is varied from 1.25 × 10^−15^ to 3.5 × 10^−16^ m^2^, and the porosity is from 0.09 to 0.13. In this study, the inlet boundary condition of AH flow is applied at the pupil which is shown in Figure 3 and the governing equation is

(10) $$\begin{array}{c} \overset{⟶}{v}=v_{o}\overset{⟶}{n}, \end{array}$$

where *v*_*o*_ is the inlet velocity related to the AH flow rate, *v*_*o*_ = AH flow rate/Inlet area . In this study, the inlet area is 0.21 × 10^−4^ m^2^ for both the young and the elder design of the human eye [23]. The age-dependent AH flow rate is shown in Table 3. The pressure at the TM outlet is equal to the episcleral venous pressure of the eye (*p* = 1200 Pa) [43]. The episcleral venous pressure is independent of aging and ranges typically from 1066 Pa to 1280 Pa [44]. In this study, the value of episcleral venous pressure has been considered to be equal to 1200 Pa and the same for the two age groups. The common boundary between the TM and AC has equal pressure across the boundary, P_AC_ = P_TM_. No-slip ($\overset{⟶}{v}=0$) boundary condition is applied at other boundaries of AC, PC, and TM [14]. The boundary conditions and the direction of the aqueous flow are shown in Figure 3. The red dashed line shows the direction of the aqueous flow. The inlet of aqueous flow is at the ciliary body, it flows through the gap between iris and lens, and finally aqueous humor outflows through the trabecular meshwork.

### 2.5. Numerical Methods

**Figure 4:**
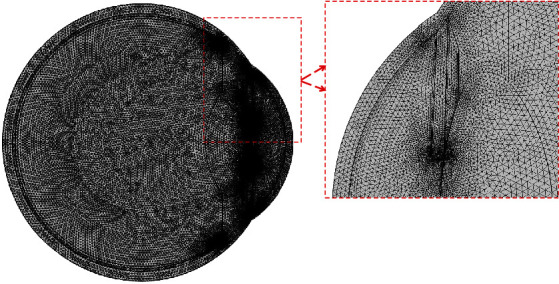
2D Mesh for FEM simulation.

Finite element method (FEM) has been applied using COMSOL Multiphysics 5.3a to evaluate the temperature profile of the human eye, pressure in the anterior chamber, and velocity profile of the aqueous humor flow in this study. Using numerical methods, like FEM, has the benefit to bring out the numerical solution of the equations governing the ocular thermo-dynamics. The solutions can be crucial to better understand the ocular temperature regulations under different conditions. To apply FEM, the developed eye model has been imported into the COMSOL Multiphysics to create a 2D mesh of triangular elements. The convergence test of the 2D mesh has been carried out to determine whether the results are independent of the mesh size. The convergence test has been evaluated for all the three dependent variables in this study: ocular temperature, aqueous velocity, and intraocular pressure. The convergence test, shown in Table 4, has been evaluated for the young eye in the horizontal orientation. Negligible differences have been observed for reducing the maximum element size from 1.04 mm to 0.155 mm. In this study, simulations of the young human eye have been carried out using a 2D mesh of 42891 triangular domain elements with a maximum element size of 0.3 mm, as shown in Figure 4. The elder human eye model has also been evaluated to be mesh convergent. The 2D mesh of the elder eye consists of 46160 triangular domain elements with a maximum element size of 0.3 mm.

The governing equations of temperature have been discretized using quadratic Lagrange in the heat transfer module of COMSOL Multiphysics. First-order Lagrange approximation has been applied for the discretization of the governing equations of velocity and pressure in the laminar flow module. To develop a steady-state solution, the discretized equations have been solved using the linear multifrontal massively parallel sparse direct solver (MUMPS). The ocular tissues have been modeled with the properties shown in Table 5. This study has been carried out to simulate two orientations of the human eye. The horizontal orientation of the eye indicates a person in a standing or sitting position, and the vertical orientation indicates supine position.

## 3. Results and Discussions

### 3.1. Temperature Profile

**Figure 5:**
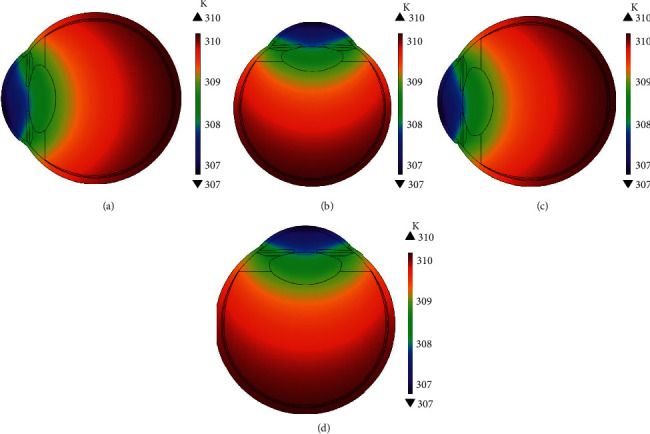
2D temperature profiles of human eye: (a, b) young and (c, d) elder human eye in the horizontal and the vertical orientations, respectively.

**Figure 6:**
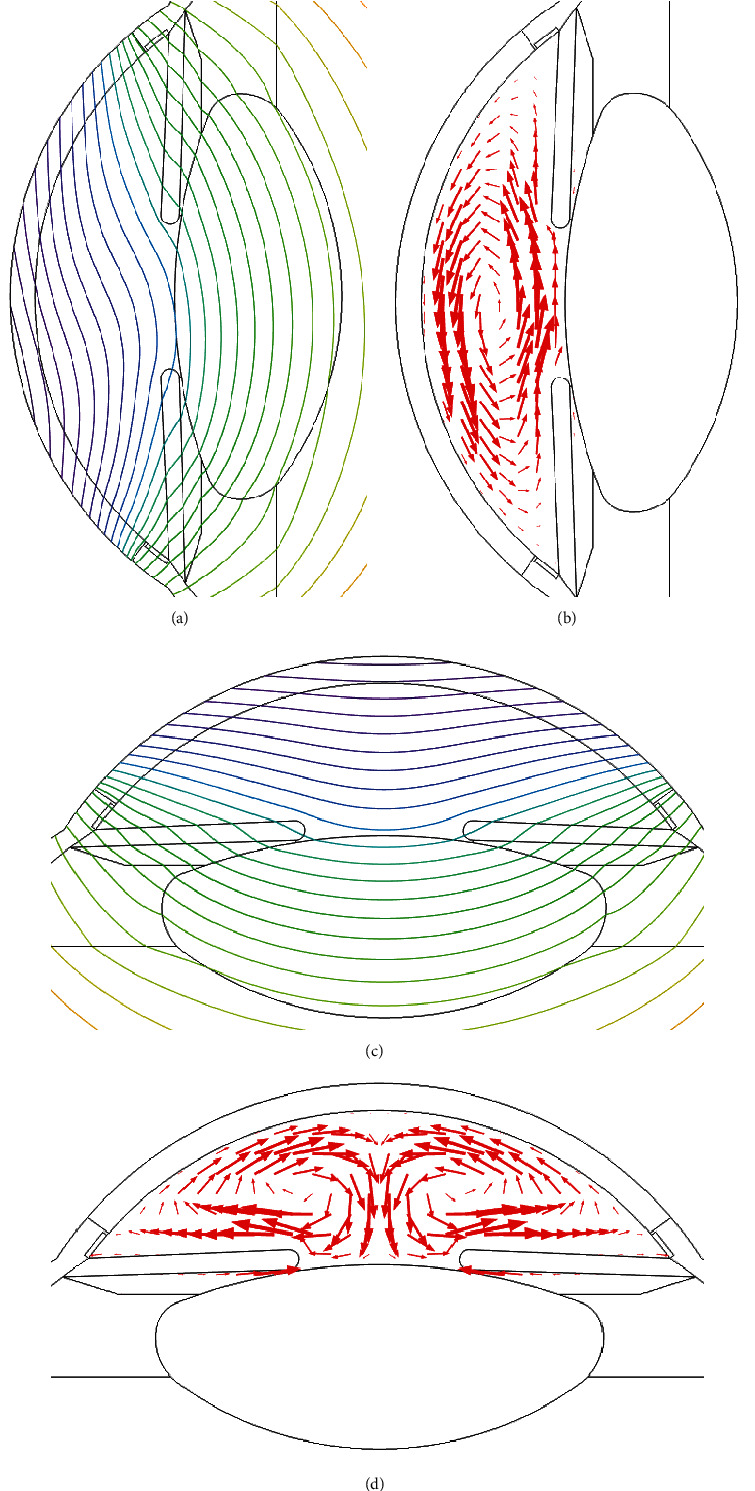
Isothermal contours and flow vectors of the young human eye: (a, b) horizontal orientation and (c, d) vertical orientation of the human eye.

The 2D temperature distributions are shown in Figure 5. The temperature profiles are warmer inside the eye being maximum of 310.15 K at sclera. The temperature decreases toward the cornea due to the heat loss at the outer corneal surface. Temperature profiles of the young and the elder human eye model in the horizontal orientation are shown in Figures 5(a) and 5(c), respectively. The temperature profiles at the vertical orientation are shown in Figures 5(b) and 5(d). The contours of temperature profiles of the young human eye are shown in Figures 6(a) and 6(c). The convective heat transfer due to the aqueous flow in the anterior chamber causes the contours to be different in the horizontal and vertical orientations of the human eye. The contours, shown in Figure 6, are asymmetric for the horizontal orientation and symmetric for the vertical orientation of the human eye. The simulated flow vectors of aqueous humor are shown in Figures 6(b) and 6(d). Aqueous flow patterns have been observed in the literature to be similar to the simulated results of this study [19].

**Figure 7:**
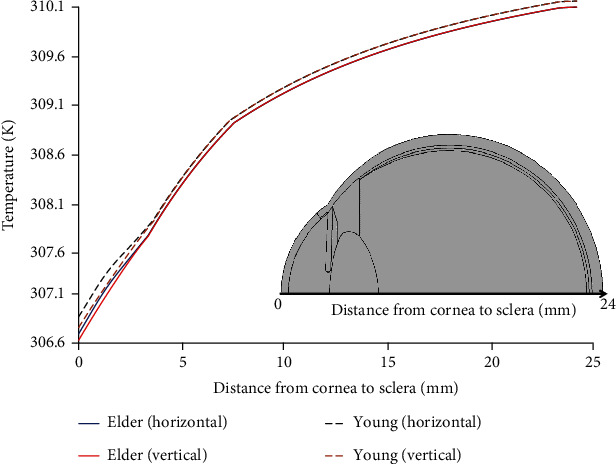
Temperature variation from the geometric center of the cornea to the sclera.

The temperature variations from the midpoint of the cornea to the sclera are shown in Figure 7. The dashed lines of Figure 7 represent the temperature of the young eye, while the solid lines represent the elder eye simulation. The minimum temperatures on these curves are at 0 mm of the *x*-axis, the geometric center of the corneal surface. The minimum values of 306.76 K and 306.88 K are observed for the young eye in the horizontal and the vertical orientations, respectively. The results of the elder eye show the minimum temperature of 306.7 K and 306.63 K in the horizontal and the vertical positions, respectively. The temperature of the elder eye is lower along the *x*-axis than the young eye in Figure 7.

**Figure 8:**
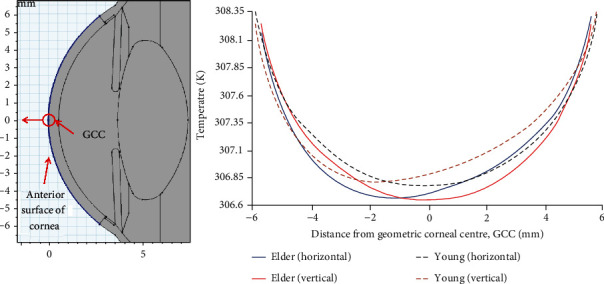
Temperature variations at the anterior surface of cornea.

The temperature variations along the surface of the cornea are shown in Figure 8. In this figure, the center (0 mm) indicates the midpoint or the geometric center of the cornea (GCC). The blue edge in Figure 8 is the anterior surface of the cornea where the temperature has been evaluated. The variations of simulated temperature profiles along the *y*-axis of eye-geometry are shown in Figure 8. Solid lines represent the elder eye model, whereas the dashed lines imply the young eye simulations. The minimum temperatures on the corneal surface of the elder groups, shown in Figure 8, are proximal to the geometric center of the cornea for horizontal orientation of the eye, while it is distant for the young eye. The magnitude of the aqueous flow velocity reduces with age due to the 2.4% decline per decade in the aqueous flow rate. This causes the convective heat transfer in the anterior chamber, caused by the aqueous flow to reduce in the elder human eye. Hence, the change in the convective heat flux is responsible for shifting the minimum point on this curve toward the GCC. Aqueous humor flow is also responsible for the symmetric temperature distributions in the vertical orientation of the eye as shown in Figure 6. The decrease in temperature of the elder eye model is observed in both simulated orientations of the eye.

**Figure 9:**
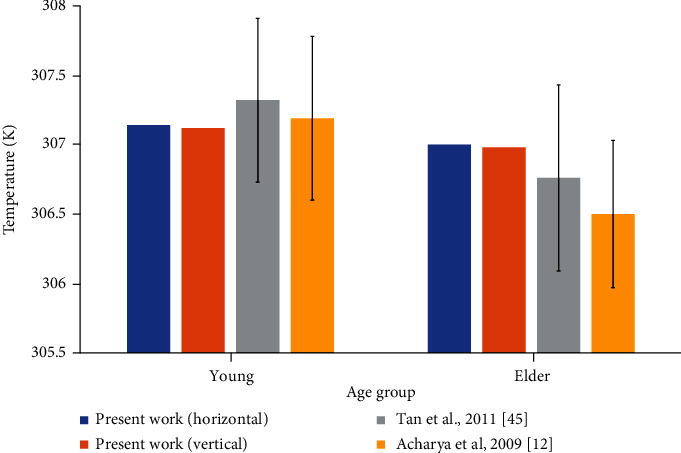
Comparison of the average temperature on the corneal anterior surface with the practical research works (the error bar represents standard deviation of practical results).

To make comparison with the recent practical studies, shown in Figure 9, the simulations of this study have been carried out at the ambient temperature, *T*_amb_ of 295.5 K. The recent practical works have measured the ocular temperature using infrared thermography. The study by Tan et al. reveals that young people have an average of 307.32 ± 0.59 K temperature on the cornea [45]. Acharya et al. showed that the average temperature on the cornea is 307.19 ± 0.59 K for young people with age below 30 [12]. Practical studies have observed decreased temperature among elder people. The people, aged above 30 years, have 306.5 ± 0.53 K of average corneal temperature as of the study in 2009 [12]. In the practical work of 2011, elder people, aged between 40 to 49 years, have 306.76 ± 0.67 K of average corneal temperature [45]. In Figure 9, the results of the young human eye of this study comply with the practical observations. Though the temperatures of the elder group in our simulation in Figure 9 are higher than the mean values of practical measurements, the results stay close to the practically observed temperatures.

The reasons for the temperature decline with aging in our study are (i) increased tear evaporation, (ii) reduced aqueous humor flow, (iii) lower metabolism in sclera, choroid, retina, and iris, and (iv) reduced blood perfusion in choroid and retina. The modeled physiological changes with aging have shown that the temperature on the corneal anterior surface changes at the rate of – 0.0075 K per year for the simulated horizontal orientation of the human eye. The rate of change of temperature is – 0.007 K per for the vertical orientation. A comparison with the recent works on ocular aging is shown in Table 6. The simulation-based ocular aging study by Samaras has modeled aging the effects of the anatomical changes and the tear evaporation rate [13]. The research by Bhandari et al. has simulated the aging changes related to ocular anatomy, tear evaporation, and aqueous fluid dynamics [14]. The present study incorporates the decline in the blood flow and the metabolic activities of ocular tissues in addition to the anatomical changes and the tear evaporation. The simulations of this study incorporate more physiological phenomena of the human eye. Therefore, the comparison of Table 6 represents that the results of this study comply more with the practical observations.

### 3.2. Intraocular Pressure

**Figure 10:**
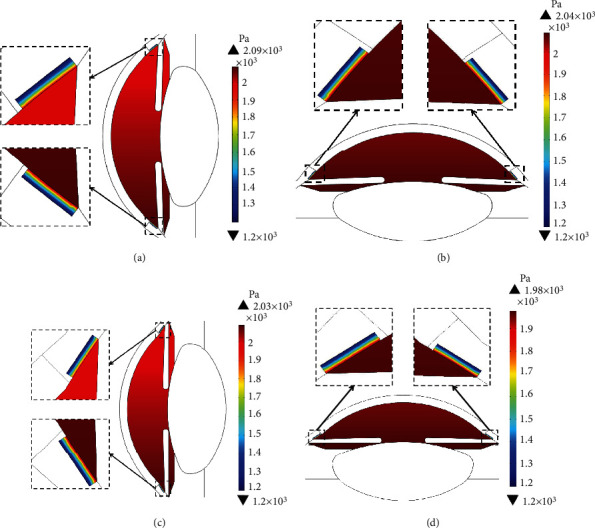
Simulated intraocular pressure (IOP) in AC and PC: (a, b) for the young eye and (c, d) for the elder eye in the horizontal and the vertical orientations of human eyes, respectively (permeability = 6.17 × 10^−16^ m^2^ and porosity = 0.105).

The simulation results of the intraocular pressure (IOP) are shown in Figure 10. The results show that the young eye has higher intraocular pressure. The higher intraocular pressure in the young eye is due to the higher aqueous humor flow rate. IOP profiles in the horizontal orientation for young and elder are shown in Figures 10(a) and 10(c), respectively. IOP in the horizontal orientation is higher in the downward direction due to the effect of gravitational acceleration. The IOP gradually decreases toward the TM outlet where the pressure is equal to 1200 Pa. The IOP profiles in the vertical orientation of the eye, shown in Figures 10(b) and 10(d), have almost a similar pressure distribution in the anterior chamber.

**Figure 11:**
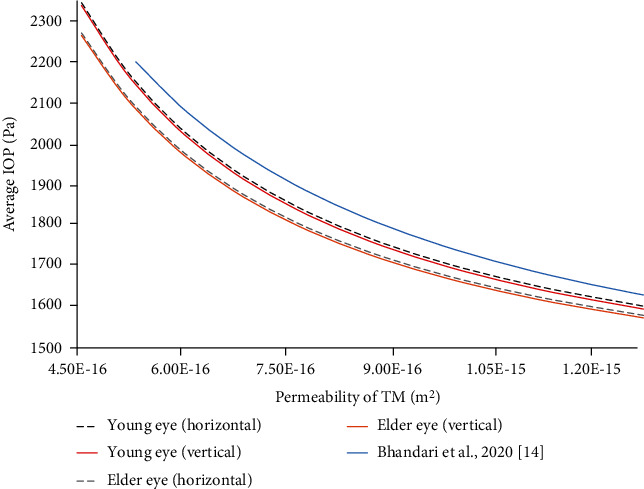
Average intraocular pressure (IOP) vs. the permeability of trabecular meshwork.

The change of intraocular pressure (IOP) with the permeability of trabecular meshwork of a healthy eye is shown in Figure 11. This figure depicts that the average intraocular pressure in the horizontal orientation is slightly higher than the vertical orientation. In comparison with the research work by Bhandari et al., the results of our study follow a similar trend of decreasing IOP with permeability but lower than the observed results of 2020 [14]. The practical studies on IOP with aging show that intraocular pressure decreases with age for men but there is no such relation for women [46]. Moreover, the IOP of healthy human adults varies between 1466.55 Pa and 2799.77 Pa [47]. The study of 2020 has applied the Stokes–Brinkman equation to model the trabecular meshwork and observed the intraocular pressure to increase with age [14]. However, results of the practical experiments, shown in Table 7, indicate that intraocular pressure either declines or does not have any relation with age. The sample sizes of the practical works are mentioned in Table 7. The practical experiments on 6043 healthy male participants have found the intraocular pressure to decline with age in [48]. Although the study in [48] has not found such a relation in the female participants, in this study, although the simulation methods do not have any defining parameters to simulate the male or female human eye, the results show the intraocular pressure to decline in the elder human eye.

### 3.3. The Velocity of Aqueous Humor Flow

**Figure 12:**
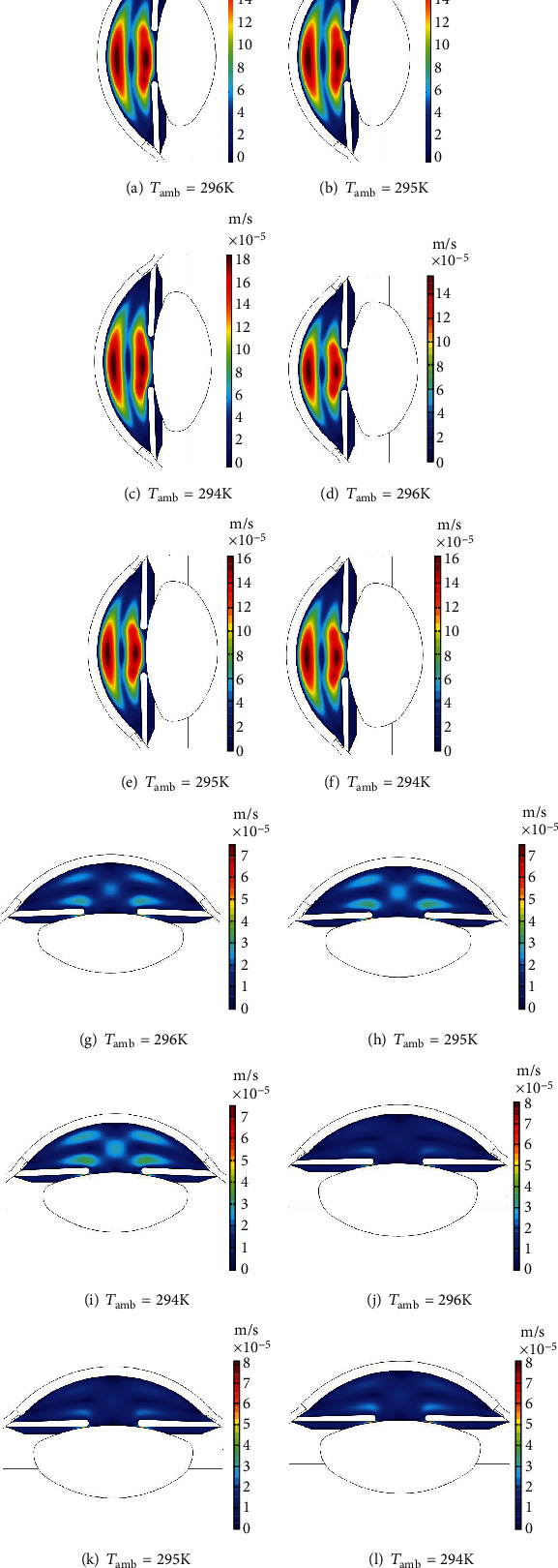
Velocity magnitude of the aqueous humor flow in the anterior and posterior chamber: (a)–(c) young eye in the horizontal position, (d)–(f) elder eye in the horizontal position, (g)–(i) young eye in the vertical position, and (j)–(l) elder eye in the vertical position.

The velocity profiles of aqueous humor (AH) flow are shown in Figure 12. The simulations have been done at the ambient temperatures, *T*_amb_, of 296 K, 295 K, and 294 K to analyze the effects of temperature on the aqueous flow magnitude. The blood temperature has been taken at 310 K for all the cases of simulations shown in Figure 12. The young eye has a maximum magnitude of 1.82 × 10^−4^ ms^−1^ in the horizontal in Figure 12(a) and 7.48 × 10^−5^ ms^−1^ in the vertical position in Figure 12(g) at the ambient temperature of 296 K. The maximum velocity for the elder eye is 1.48 × 10^−4^ms^−1^ in the horizontal in Figure 12(d) and 7.51 × 10^−5^ ms^−1^ in the vertical position in Figure 12(g). The velocity magnitude increases with decreasing the ambient temperature as shown in Figure 12. Heat transfer through convection increases with the decline in the ambient temperature causing the increase of velocity magnitude. The velocity profile has a lower magnitude in the case of the elder eye due to the low AH production rate. The comparison of the horizontal and the vertical velocity magnitude, shown in Table 8, implies that the results of our simulation are in the order of the observed results in the literature. However, the differences in the velocity magnitude in this table are due to the variation of research methodologies of different studies. The methodologies of these studies are different to apply the boundary conditions of the aqueous humor flow and the human eye model used in the simulations. No inlet and outlet boundary conditions have been applied in [19, 20]. The study in [14] has applied the inlet boundary at the ciliary body and the outlet at the trabecular meshwork of the human eye.

## 4. Conclusion

The study has incorporated the anatomical and physiological changes of the human eye with aging to model the thermofluid dynamics of the human eye. Four physiological changes have been included in the simulation: metabolism, blood perfusion, tear evaporation, and aqueous humor flow. The temperature change of – 0.0075 K per year in the horizontal position and – 0.007 K per year in the vertical position of the human eye have been observed in the study due to the simulated physiological effects. Lower intraocular pressure has been observed in the simulation for the elder eye due to the reduction in aqueous humor production rate by 2.4% per ten years. The velocity of aqueous flow in the simulation has been higher in the young human eye simulation due to the higher aqueous flow rate.

There are some deviations of the simulated results from the practical ones mentioned in various literature. This deviation is due to the unavailability of physiological data for some ocular tissues. The CAD model used in this work tends to replicate the human eye as closely as possible with acceptable accuracy. Parameters like metabolism, tear evaporation, blood flow, and dynamics of aqueous humor are included in this model which can be altered to model diseased conditions of the eye. Moreover, this model depicts the variation of eye tissues with age; hence, this model would also help in studying the progression of ocular diseases with age. As ocular studies are quite tedious and experimenting on live subjects requires accuracy and sophistication, this detailed mathematical model of the human eye could be a significant contribution in observing the progression and early detection of various ocular diseases.

## Figures and Tables

**Table 1:** Recent research works on the aqueous humor flow simulation.

Reference	Simulation features	AH inlet	AH outlet
Ooi and Ng [19]	Effects of AH flow on eye temperature, 2D–7 HR eye model	No inlet	No outlet
Karampatzakis and Samaras [20]	Effects of AH flow on eye temperature, 3D–6 HR eye model	No inlet	No outlet
Karampatzakis and Samaras [21]	Effects of AH flow on eye temperature, 2D–7 HR eye model	Pupil	TM
Samaras [13]	Modeling ocular aging using 2D–6 HR eye model	No inlet	No outlet
Tiang and Ooi [22]	Alterations of eye temperature for EM waves, 3D–7 HR eye model	No inlet	No outlet
Loke et al. [23]	AH outflow analysis for drug delivery, 3D–8 HR eye model	CB	TM, applied Stokes–Brinkman equation
Bhandari et al. [14]	Ocular aging, drug delivery simulation, 2D–9 HR eye model	CB	TM, applied Stokes–Brinkman equation
Zarei et al. [10]	Effects of glasses on ocular temperature, 2D–7 HR eye model	No inlet	No outlet
Present study	Effects of aging on ocular temperature, AH flow, and IOP, 2D–11 HR eye model	CB	TM, Darcy's law

HR: homogenous region; AH: aqueous humor; CB: ciliary body; IOP: intraocular pressure; TM: trabecular meshwork.

**Table 2:** Age-related changes in the CAD model of human eye [24, 25].

Age group	Anterior chamber thickness (ACT)	Lens thickness (LT)	Lens width (LW)	The radius of lens anterior surface	Radius lens posterior surface
21–30 (young)	3.1 mm	3.71 mm	9 mm	11.188 mm	6.99 mm
41–50 (elder)	2.84 mm	4.18 mm	9.2 mm	10.312 mm	6.84 mm

**Table 3:** Simulation parameters of the physiological changes with age.

Parameter	Young	Elder	Reference
Tear evaporation rate	27 Wm^−2^	41.8 Wm^−2^	[30]
Aqueous flow rate at ciliary body	4.67 × 10^−11^m^3^s^−1^	4.33 × 10^−11^m^3^s^−1^	[14]
Blood perfusion rate in choroid	0.01298 ml(ml^−1^s^−1^)	0.01038 ml(ml^−1^s^−1^)	[32]
Blood perfusion rate in iris	9.26 × 10^−3^ml(ml^−1^s^−1^)	7.41 × 10^−3^ ml(ml^−1^s^−1^)	[33]
Basal metabolism in choroid, retina and sclera	22000 Wm^−3^	17600 Wm^−3^	[34]
Basal metabolism in iris	10000 Wm^−3^	8000 Wm^−3^	[34]

**Table 4:** Mesh convergence test of the study.

Number of domain elements	Maximum element size (mm)	Minimum temperature of cornea (K)	Maximum aqueous velocity in the anterior chamber (ms^−1^)	Intraocular pressure (Pa)
44695	0.155	306.8057053	1.82 × 10^−4^	1951.129377
42891	0.3	306.8056656	1.81 × 10^−4^	1950.852822
42189	0.647	306.8056687	1.81 × 10^−4^	1951.176187
41317	0.809	306.8056831	1.81 × 10^−4^	1951.165463
40951	1.04	306.8056675	1.82 × 10^−4^	1951.212902

**Table 5:** Properties of the thermodynamics simulation of human eye [21].

Eye tissue	Thermal conductivity (Wm^–1^K^−1^)	Heat capacity at constant pressure (JKg^–1^K^−1^)	Density (Kgm^−1^)
Cornea	0.58	4178	1050
AC and PC	0.58	3997	996
Lens	0.4	3000	1050
Iris	1.0042	3180	1100
VH	0.603	4178	1000
Sclera	1.0042	3180	1100
CB	0.498	3340	1040
Retina	0.565	3680	1039
Choroid	0.53	3840	1060
TM	1.004	3180	1100

**Table 6:** Comparison with the recent works on ocular aging.

Reference	Age (year)	Mean temperature on cornea	Study of aging effects
Simulation work [13]	20–29	308.3 K	Anatomy and tear evaporation
	40–49	308.2 K	
Simulation work [14]	20–29	307.6 K	Anatomy, tear evaporation, and aqueous humor flow
	40–49	307.1 K	
Present work	21–30	307.14 K	Anatomy, tear evaporation, aqueous humor flow, blood flow, and metabolism
	41–50	307 K	
Practical work [45]	18.9 ± 0.8	307.32 ± 0.59 K	Practical study using infrared thermography on 30 young and 30 elder healthy participants
	47 ± 8.4	306.76 ± 0.67 K	

**Table 7:** Relation between aging of healthy eye and intraocular pressure (IOP).

Reference	Age group (years)	IOP (Pa)	Remarks
Torris et al. [49]	20–30	1959.84 ± 346.64	51 young and 53 elder healthy people
	Age ≥60	1906.51 ± 346.64	
Kaufman [50]	20–30	1973.17 ± 333.31	38 young and 25 elder healthy people
	Age ≥60	1986.503 ± 386.63	
Lin et al. [48]	20–39	1893.18	Averages of 6043 healthy male participants of age 21–79 years old)
	40–59	1839.85	
	60–79	1666.53	
2021 present study	21–30	1611.8 to 2557.9	Simulation results
	41–50	1583.1 to 2463.5	

**Table 8:** Comparison of the maximum velocity magnitude of AH flow of young human eye.

Eye position	Present study	Bhandari et al. [14]	Karampatzakis and Samaras [20]	Ooi and Ng [19]
Horizontal	1.82 × 10^−4^ ms^−1^	5.75 × 10^−4^ ms^−1^	3.36 × 10^−4^ms^−1^	1.03 × 10^−4^ ms^−1^
Vertical	7.51 × 10^−5^ ms^−1^	4.26 × 10^−4^ ms^−1^	1.7 × 10^−5^ms^−1^	2.78 × 10^−5^ ms^−1^
*T*_amb_	296 K	296 K	296 K	298 K

## Data Availability

The data used to support the findings of this study are included in the article.
